# Purinergic signaling regulates mobilization of hematopoietic stem cells

**DOI:** 10.18632/oncotarget.26290

**Published:** 2018-11-16

**Authors:** Mateusz Adamiak, Ahmed Abdel-Latif, Mariusz Z. Ratajczak

**Affiliations:** Stem Cell Institute at James Graham Brown Cancer Center, University of Louisville, Louisville, KY, USA; Department of Regenerative Medicine Warsaw Medical University, Warsaw, Poland

**Keywords:** complement cascade, purinergic signaling, extracellular nucleotides, hematopoietic stem cells, stem cell mobilization

Hematopoietic stem progenitor cells (HSPCs) reside in bone marrow (BM) niches and their retention is facilitated by receptors associated with membrane lipid rafts, α-chemokine receptor CXCR4 and integrin receptor VLA-4, expressed on HSPCs that interact with their specific ligands, expressed in stem cell niches - stromal derived factor-1 (SDF-1) and vascular cell adhesion molecule 1 (VCAM-1), respectively. HSPCs are mobilized from the BM into peripheral blood (PB) in response to: i) systemic or local infection; ii) stress related to tissue organ injury; or iii) hypoxia [1]. Innate immunity plays a pivotal role in this process and optimal mobilization requires activation of Gr-1^+^ leucocytes that secrete proteolytic and lipolytic enzymes as well as danger associated molecular pattern molecules (DAMPs)—leading to the activation of complement cascade (ComC) and release of ComC cleavage fragments (C5a, C5b-C9) required for optimal egress of HSPCs to PB [2, 3]. Mobilization is also collaterally affected by the activation of coagulation cascade (CoaC), as both of these ancient proteolytic cascades may cross-activate each other [4].

The cytokine granulocyte colony stimulating factor (G-CSF) and the small molecular CXCR4 antagonist AMD3100, also known as Plerixafor induce the forced egress of HSPCs into PB [1]. Both of these drugs are explored in clinical settings during pharmacological mobilization to harvest HSPCs from PB by leucopheresis as a source of cells for hematopoietic transplants. Since some patients do not respond efficiently to currently recommended mobilization protocols and are deemed poor mobilizers, it is important to develop more efficient mobilization strategies in order to obtain the required number of HSPCs to perform successful transplantation.

There are described several molecular and cellular events triggered in the BM microenvironment after the administration of mobilization promoting agents (G-CSF or AMD3100) [1]. Our group recently reported that mobilization of HSPCs from BM into PB is tightly regulated by purinergic signaling that is a form of extracellular signaling mediated mainly by secretion from the cells adenosine triphosphate (ATP) and its metabolite adenosine [5]. Both these extracellular nucleotides (EXN) interact with purinergic receptors from P1, P2X, and P2Y receptor families expressed on cells, that are among the most abundant receptors in living organisms. ATP and its degradation product nucleoside adenosine, have been reported to regulate the migration of normal and malignant hematopoietic cells. Moreover, both these mediators have opposite effects on the mobilization process [5]. As we recently demonstrated, while ATP triggers the mobilization of HSPCs from BM into PB, adenosine inhibits it by acting as a negative feedback molecule [5].

Adenosine is generated in the extracellular space in a process of ATP degradation by the cell surface expressed ectonucleotidases CD39 and CD73. Thus, administration of ATP or inhibition of CD73 or CD39 ectonucleotidases by small molecule antagonists may provide the basis for new and more efficient mobilization strategies by decreasing the concentration of adenosine in extracellular space [5]. This in fact we have currently confirmed in our laboratories by employing such compounds in the murine model of HSPCs mobilization (manuscript in preparation).

**Figure 1:**
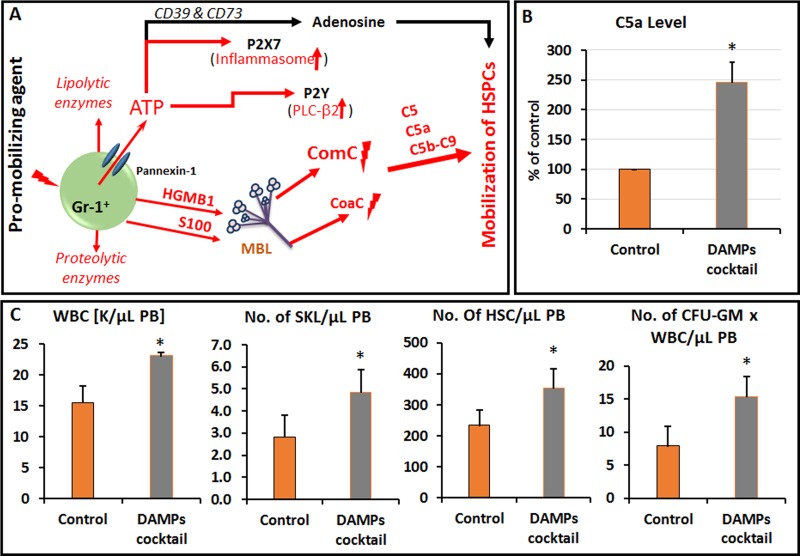
**A.** Interplay between purinergic signaling and ComC activation during mobilization of HSPCs. Pro-mobilizing agent (e.g., G-CSF) activates Gr-1+ cells to secrete proteolytic and lipolytic enymes as well as several DAMPs including: ATP, HGMB1 and S100. While HGMB1 and S100 proteins activate complement cascade (ComC) in MBL-dependent pathway, ATP interacts with P2X7 and P2Y purinergic receptors on hematopoietic cells what leads to activation of inflammasome and release of PLC-β2. Both inflammasome and PLC-β2 promote the effective mobilization. At the same time ATP is processed by CD39 and CD73 ectonucleotidases to adenosine, that inhibits the mobilization process by upregulating in HSPCs heme oxygenase-1 (HO-1). ComC activated in MBL-dependent pathway together with CoaC is crucial in the executing egress of HSPCs from BM into PB. Pathways promoting mobilization are shown by red arrows, and adenosine inhibitory pathway by black arrow. **B.** Activation of complement cascade and release of C5a after administration of DAMPs (ATP + HGMB1 + S100). C5a level has been measured in PB by employing sensitive ELISA assay. *p < 0.001. C. Administration of DAMPs enhances mobilization of HSPCs in mice. Mononuclear cells (MNCs) were isolated from WT mice mobilized for 6 days with G-CSF alone (control) and 6 days with G-CSF + cocktail of DAMPs (ATP + HGMB1 + S100). The numbers of WBCs, SKL (Sca-1+ /c-kit+ /Lin− ) cells, HSCs (Sca-1+ /CD45+ /Lin− ), and CFU-GM clonogenic progenitors were evaluated in PB. Results from two independent experiments are pooled together. *p < 0.001.

The molecular explanation of involvement of purinergic signaling in HSPCs mobilization is explained by the initial release of ATP from the Gr-1^+^ cells in the BM microenvironment in response to pro-mobilizing agents (e.g., G-CSF) (Figure 1A). ATP is released from the cells through the cell membrane channel known as pannexin-1 and in the extracellular space activates P2X7 purinergic receptor present on hematopoietic cells [5]. This process leads to the intracellular activation of inflammasome. In support of this we noticed that blockage of pannexin-1 channel by employing blocking peptide or depletion of P2X7 on bone marrow hematopoietic cells significantly decreases mobilization of HSPCs [5]. Based on these observations, ATP release links purinergic signaling with activation of ComC and CoaC to trigger egress of HSPCs from BM into PB.

ATP secreted into extracellular space form Gr-1^+^ BM cells is one of danger associated molecular pattern molecules (DAMPs). Gr-1^+^ cells also secrete other DAMPs including the chromatin-associated protein high-mobility group box 1 (HMGB1) and multigenic family of calcium modulated S100 proteins (Figure 1A). DAMPs proteins are recognized by mannan binding lectin (MBL) that is pattern recognition receptor circulating in peripheral blood. This leads to MBL-dependent activation of ComC and CoaC and the release of C5 cleavage fragments C5a and C5b-C9 required for optimal egress of HSPCs from BM into PB. In fact as we reported mice that do not express MBL or C5 are poor mobilizers [3, 6, 7]. As it is shown in Figure 1, administration to mice of a cocktail of DAMPs including ATP, HMGB-1 and S100 induces activation of the ComC as evidenced by an increase in serum level of C5a (Figure 1B) and enhances mobilization of HSPCs (Figure 1C).

ATP as DAMP member plays an important additional role by activating P2X7-dependent manner inflammasome as well as by involving P2Y receptors releases from Gr-1^+^ cells phospholipase-2 beta (PLC-β2). This lipolytic enzyme disintegrates membrane lipid rafts on HSPCs that contain BM-retention receptors CXCR4 and VLA4 and thus facilitates release of HSPCs from BM stem cell niches [8]. This pro-mobilizing effect of ATP is negatively regulated as we recently noticed by extracellular adenosine. These opposite role of ATP and adenosine is explained by different effects on intracellular expression of heme oxygenase-1 (HO-1), that is a negative regulator of migration and mobilization of HSPCs [9]. Accordingly, while ATP inhibits HO-1, adenosine upregulates expression of this enzyme in HSPCs [5].

In conclusion, our most recent data shed more light on mobilization of HSPCs and involvement in this process of purinergic signaling including ATP, adenosine, other DAMPs and supports a crucial role of MBL pathway on ComC activation (Figure 1C). Since ~10% of normal patients are poor mobilizers of HSPCs, modulation of purinergic signaling in the BM microenvironment may lead to better mobilization of HSPCs. Our observations are also highly relevant for other processes in which an increase in stem cell trafficking occurs, such as for example in stress related to infection, tissue/organ injury, or strenuous exercise - as in all these processes is observed an interplay between purinergic signaling and activation of ComC [1,7]. Further studies are also required to assess effect of purinergic signaling on homing and engraftment of transplanted HSPCs [10].
